# The Consequences of Coronavirus-Induced Cytokine Storm Are Associated With Neurological Diseases, Which May Be Preventable

**DOI:** 10.3389/fneur.2020.00745

**Published:** 2020-07-24

**Authors:** Ole Isacson

**Affiliations:** Department of Neurology (Neuroscience), Harvard Medical School, McLean Hospital, Belmont, MA, United States

**Keywords:** pandemic, viral infection, toll-like receptors, Parkinson's disease (PD), interleukin-1 receptor antagonist (IL-1 ra), COVID-19, SARS-CoV-2

In the wake of the Spanish flu pandemic (starting 1918) over a million people who survived the viral infection subsequently developed Parkinson's disease (PD); acquiring the so called post-encephalitic PD (PEPD). This was dramatized in the movie “Awakenings” (1990) with Robin Williams as the doctor and Robert De Niro as the patient. In fact, early treatment evidence of the currently used drug L-dopa for PD was obtained in such PEPD patients. To give a perspective on the magnitude of this problem, in the 15 years leading up to 1940, approximately 50% of all PD cases were diagnosed as PEPD. Such PEPD cases are now rare, while there are other flu viruses that can generate a small number of PEPD cases, for example, the so-called Japanese flu; with clear evidence of selective neuroinflammatory responses in cells that are vulnerable to develop PD pathology in the human brain (1). While we now know that aging, environment and genetic factors combined drive most of the PD risk—there is reason to believe that neuroinflammation plays a role in the development of disease (2).

We have tried to understand this general risk factor for developing PD and other neurodegenerative diseases in the context of cytokine storm (1–4). Cytokine storm [cytokines are molecules that signal to other immune cells to activate an immune attack by producing antibodies from white blood cells (B cells) or killing infected cells by T cells] can represent a condition in which the immune system battles infections, even successfully, but over time this immune activation reaches such proportions that tissues no longer containing pathogens (such as viruses) will continue to be attacked in a non-specific way. This is relevant to understanding the current coronavirus outbreak, in particular as it has been reported that individuals who have been cleared of coronavirus infection (COVID-19) then die several days later in what appears to be a fulminant systemic inflammation in part caused by excessive cytokine elevations. In research starting in 2007 (3, 4) we developed a mouse model system to infuse synthetic viral RNA (Poly I:C) in brain regions that typically are vulnerable in PD (4). Such synthetic DNA does not replicate and does not produce an infection but act on the same immune stimulatory receptors (Toll-like 3 receptors) as viruses do on cells. We found that such viral-like RNA could elicit a progressive increase in cytokines in the vulnerable brain regions over 7–14 days without overtly killing cells like the midbrain dopaminergic neurons, which would have led to the parkinsonian syndrome. However, when we added oxidative stress in the vulnerable brain region of PD (a double-hit model), by infusing mild neurotoxins at the peak of this cytokine activation (12 days after the viral RNA exposure), the brain dopaminergic neurons died at a much higher rate than without the cytokines—in this way representing a model of PEPD. We studied the specific interleukins (which are cytokines) that peaked around 12–14 days. We found interleukin 1-beta (IL1b) to be one of the most highly expressed cytokines in the vulnerable brain regions 12 days after the viral-like activation. We therefore did an experiment where we blocked IL1b systemically (using an IL1b receptor antagonist) in research model rodents and found that this prevented many of the vulnerable dopamine neurons from degenerating and dying. The IL1b successful blockade of the deleterious effects of excessive cytokine induction and sparing of brain cells in our studies (3, 4), is potentially relevant to similar approaches now being tested for blocking Covid-19 induced damage in lung-tissues by administration of IL6 antibodies to patients (FDA Phase 3; “Covacta” trial of “Actemra,” and a FDA Phase 2 trial of “Kevzara”).

It may be of importance to revisit these findings in the context of how brain immune microglia and neurons can be activated in specific ways by viral RNA, independently or in the context of an infection that causes the flu like symptoms and disease (1, 5). In particular, it highlights that recording evidence of excessive cytokine activations in patients in the wake of successful elimination of propagating viruses, such as coronaviruses, could be very informative for anticipating future cases of certain neurological diseases.

Many Covid-19 patients present early in the viral disease with a loss of smell, which is a neurological manifestation. Loss of smell is a well-known antecedant signal for developing Parkinson's disease and other neurodegenerative diseases (6). In rare instances of Covid-19 acute cases, brain scans (MRI and CT) have also shown acute brain pathology and brain damage related to hemorrhage (7), potentially related to inflammation. Clearly, many of the neurological effects of COVID-19 may be self-limiting, and resolve without any marked damage to the brain and peripheral nervous system, as was likely the case in the Spanish Flu Pandemic 1918-1920 (but see above mentions of marked increases in PD cases -PEPD - in the years following up to 1940). Nevertheless, chronic and excessive inflammation is likely present also in idiopathic-sporadic PD given that epidemiological studies show reduced or delayed incidence of PD in users of non-steroidal anti-inflammatory substances (ibuprofen) (8), and re-analysis of data from some patients treated with TNF alpha antibodies show marked reduction in the incidence of PD (9). Given these findings, it is important to consider collecting data, analyzing and reanalyzing results from Covid-19 cases in the near and distant future, including neurological signs. Such observations may lead to neuroprotective and preventive treatments. With such data available, physicians may advise patients to use anti-inflammatory drugs (as in recommendations for heart disease risk reduction) or determine the particular cytokine-to patient profiles that represent high risk for neurodegenerative diseases, and be able to recommend treatments in the post-viral recovery phase in the severe Covid-19 patients.

Finding the specific cytokines that cause to post-viral systemic and local inflammation that leads to serious disease and even death in susceptible individuals may allow interventions to prevent serious neurological disease outcomes (1). Medicines and interventions that reduce activation of toll-like receptors or block cytokine peaks – correctly applied after the body has cleared the acute viral infections such as Covid-19 – should be investigated further.

## Author Contributions

The author confirms being the sole contributor of this work and has approved it for publication.

## Conflict of Interest

The author declares that the research was conducted in the absence of any commercial or financial relationships that could be construed as a potential conflict of interest.
